# Guest Editor's Message

**DOI:** 10.4103/0972-9941.27718

**Published:** 2006-09

**Authors:** Pradeep Chowbey

**Affiliations:** President - Asia Pacific Hemia Society Chairman Minimal Access Surgery & Bariatric Surgery Centre, Sir Ganga Ram Hospital, New Delhi, India

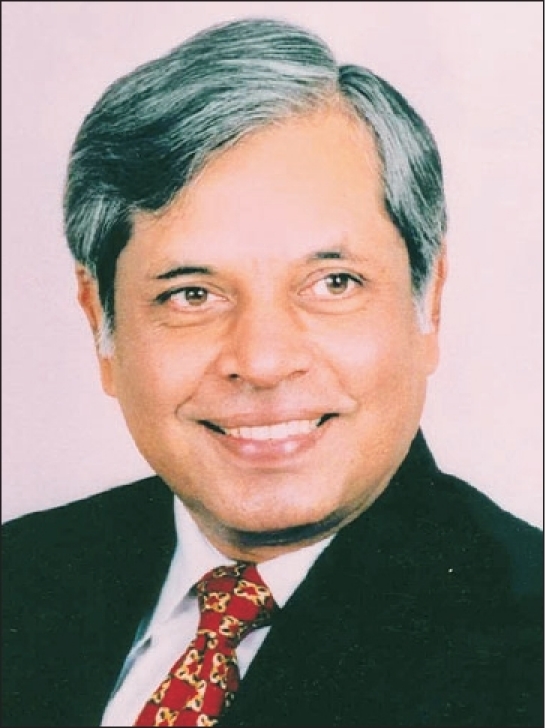



Dear Colleagues,

I am pleased to welcome you all on the occasion of the Second Congress of the Asia Pacific Hernia Society at New Delhi, India scheduled between 6–8 October 2006. I am convinced that this Hernia Congress would be a landmark event in the present era of increasing specialization.

Hernia repair is one of the most commonly performed surgical procedures by a practising surgeon today. A hernia can present at different ages in different sites at varying stages. Indeed, a surgeon practising hernia repairs has to necessarily possess a wide array of surgical repertoire and technique. There have been numerous exciting developments and products on offer to the hernia Surgeon, especially in the last two decades. The Scientific programme was designed to incorporate most of the innovations and breakthrough to keep us all abreast of current thought in herniology.

We are extremely fortunate to have been propped up by Association of Surgeons of India (ASI) and Indian Association of Gastrointestinal Endosurgeons (IAGES). The substance and essence of Indian surgery is represented between these two giant organizations and we are grateful for their support.

The Asia Pacific region is home to more than half of the World populace and is remarkable for its diversity in terms of nationalities, race, creed and ethnicities. With the formation of the Asia Pacific Hernia Society, a glaring lacuna has been plugged in the World of Hernia, with the prior existence of the American Hernia Society and European Hernia Society. As President of Asia Pacific hernia Society, I strongly encourage you to become members of APHS. This would enable you to tap expertise of not only APHS but also the American and European Hernia Societies. The Hernia Journal would be available at discounted rates as also registration to congresses of all three hernia societies.

I foresee lively brainstorming during our scientific sessions (as most of us have hard opinions on hernia) and great camaraderie in our social events.

